# Protocol for a randomized controlled study examining the role of rapid eye movement sleep in fear-related mechanisms: rapid eye movement fragmentation and fear inhibition in adults with insomnia disorders before and after cognitive behavioral therapy for insomnia

**DOI:** 10.1093/sleepadvances/zpad030

**Published:** 2023-08-03

**Authors:** Vivien Vuong, Alix Mellor, Victoria B Risbrough, Bei Bei, Sean P A Drummond

**Affiliations:** School of Psychological Sciences, The Turner Institute for Brain and Mental Health, Monash University, Clayton, VIC, Australia; School of Psychological Sciences, The Turner Institute for Brain and Mental Health, Monash University, Clayton, VIC, Australia; Department of Psychiatry, University of California San Diego, La Jolla, CA, USA; Centre of Excellence for Stress and Mental Health, VA San Diego Healthcare System, La Jolla, CA, USA; School of Psychological Sciences, The Turner Institute for Brain and Mental Health, Monash University, Clayton, VIC, Australia; School of Psychological Sciences, The Turner Institute for Brain and Mental Health, Monash University, Clayton, VIC, Australia

**Keywords:** Insomnia, REM sleep, fear inhibition, fear extinction, safety signal learning, fear-potentiated startle, cognitive behavioral therapy for insomnia (CBT-I)

## Abstract

Insomnia confers a 2.5-to-3-fold risk of developing posttraumatic stress disorder (PTSD) after a traumatic event. The mechanism underlying this increased risk, however, remains unknown. We postulate insomnia may contribute to PTSD by disrupting rapid eye movement (REM) sleep, as REM sleep disruption has been shown to impair fear inhibitory processes, which are central to the natural recovery from trauma. To test this hypothesis, the following protocol aims to: (1) examine the relationship between REM sleep and fear inhibition in insomnia, and (2) examine whether reducing REM fragmentation by treating insomnia, in turn, improves fear inhibition. Ninety-two adults with Insomnia Disorder will be block randomized (1:1; stratified by sex) to an active treatment (7 weekly sessions of Cognitive Behavioral Therapy for Insomnia (CBT-I) via telehealth) or waitlist control condition. REM sleep (latent variable derived from REM %, REM efficiency, and REM latency) and fear inhibition (i.e. safety signal and extinction recall) will be assessed pre- and post-treatment in a 4 night/3 day testing protocol via at-home polysomnography and the fear-potentiated startle paradigm, respectively. Fear extinction recall will serve as the primary outcome, while safety signal recall will serve as the secondary outcome. In summary, this study aims to test an underlying mechanism potentially explaining why insomnia greatly increases PTSD risk, while demonstrating an existing clinical intervention (CBT-I) can be used to improve this mechanism. Findings will have potential clinical implications for novel approaches in the prevention, early intervention, and treatment of PTSD.

**Statement of Significance**

PTSD affects more than 200–288 million people worldwide, and this number is only expected to increase following ongoing conflicts and increasing climate-driven natural disasters. Identifying effective ways to prevent PTSD and intervene early in the development of this disorder is thus extremely timely. Insomnia is a major risk factor for the development of PTSD. Importantly, unlike most known risk factors for PTSD, insomnia is modifiable with evidence-based treatments, making it a prime potential target for intervention. Despite this, no studies to date have directly tested the mechanism underlying insomnia’s contribution to PTSD. This lack of knowledge limits our ability to reduce the incidence of PTSD and is the critical advance our study aims to generate.

## Introduction

Global prevalence estimates of posttraumatic stress disorder (PTSD) in the wake of the coronavirus disease 2019 pandemic are approximately 17%, with even higher rates in professions at high risk of trauma exposure [1]^(p19)^. Individuals with PTSD use more healthcare resources than other psychiatric disorders [2, 3] and experience significantly reduced quality of life [4, 5]. PTSD is characterized by an impairment in the ability to inhibit fear responses to previous threat cues in safe environments. This ability is termed fear inhibition and involves two processes: fear extinction (i.e. learning to inhibit previously learned fear responses to cues no longer predicting threat), and safety signal learning (i.e. learning to identify environmental cues actively predicting the absence of threat) [6]. Fear inhibition is central to the natural recovery from trauma, and a fundamental mechanism in the development, maintenance, and treatment of PTSD [7]. Although evidence-based treatments for PTSD are available, many do not seek treatment and response rates in those that do average only approximately 67% [8, 9]. Therefore, there is a clear need to identify modifiable factors which influence the onset of PTSD in order to promote resilience and improve recovery rates.

Insomnia is one such modifiable risk factor, occurring in as many as 70%–90% of individuals with PTSD [10, 11]. Studies in both civilian and military samples suggest insomnia increases the chances of developing PTSD following trauma by 2.5–3 times [12, 13] and exacerbates PTSD symptoms in those that do [14]. Insomnia is also associated with increased conditioned fear; however, effects on fear inhibition recall are unclear [15–18]. While it is clear, then, having insomnia increases the risk of developing PTSD, the mechanism underlying this increased risk remains unknown.

Evidence from both human and animal studies suggests REM sleep plays an important mechanistic role in fear inhibition. Experimental disruption of REM sleep has been shown to impair the recall of both extinction and safety learning [19–23]. Importantly, relationships between REM sleep and fear inhibition have also been found in individuals with PTSD [24].

Given that disrupted REM sleep is a hallmark of insomnia [25], insomnia may be increasing the risk of PTSD because it is associated with the type of REM sleep disruption shown to impair fear inhibition. For example, chronic insomnia has been associated with reduced minutes and percentage of time spent in REM sleep, relative to healthy controls [26]. Furthermore, these same REM measures show significant improvements following treatment with cognitive behavioral therapy for insomnia [27]. This reversal of REM disruptions with treatment suggests the daytime consequences of disrupted REM sleep, such as impaired fear inhibition, may also be reversed.

### Current study

As outlined above: (1) impaired fear inhibition underlies the development and maintenance of PTSD, (2) REM sleep plays a mechanistic role in fear inhibition, and (3) insomnia is characterized by the very type of REM disruption implicated in impaired fear inhibition. Therefore, insomnia may be a potent risk factor for developing PTSD because disrupted REM sleep caused by insomnia may interfere with fear inhibition processes that are critical for trauma recovery. The current study will test this proposed mechanism in a group of adults with Insomnia Disorder undergoing an experimental fear conditioning and fear inhibition paradigm. The Aims and hypotheses of this study are:

*Aim 1:* Examine the contribution of REM sleep to fear inhibition in insomnia disorder.

Hypothesis 1. Greater REM fragmentation at night will be associated with lower next-day fear inhibition, operationalized as poorer recall of extinction (primary outcome) and safety signal (secondary outcome) learning.

*Aim 2:* Examine whether improving REM sleep by treating insomnia subsequently improves measures of fear inhibition.

Hypothesis 2(a). Fear inhibition will show greater improvements following an active insomnia treatment, relative to a waitlist control (i.e. significantly stronger recall of extinction (primary) and safety signals (secondary) in the active treatment group).

Hypothesis 2(b). Improvements in REM sleep (i.e. decreased fragmentation) will mediate the treatment effect on improvements in fear inhibition (primary outcome: extinction recall; secondary outcome: safety signal retention). To test the specificity of the hypothesized REM mediation effect, we will also test the mediating roles of slow wave sleep (N3%) and the insomnia severity index (ISI) in the treatment-fear inhibition relationship. We hypothesize both of those factors will be nonsignificant as mediators.

## Methods

**Figure 1.**
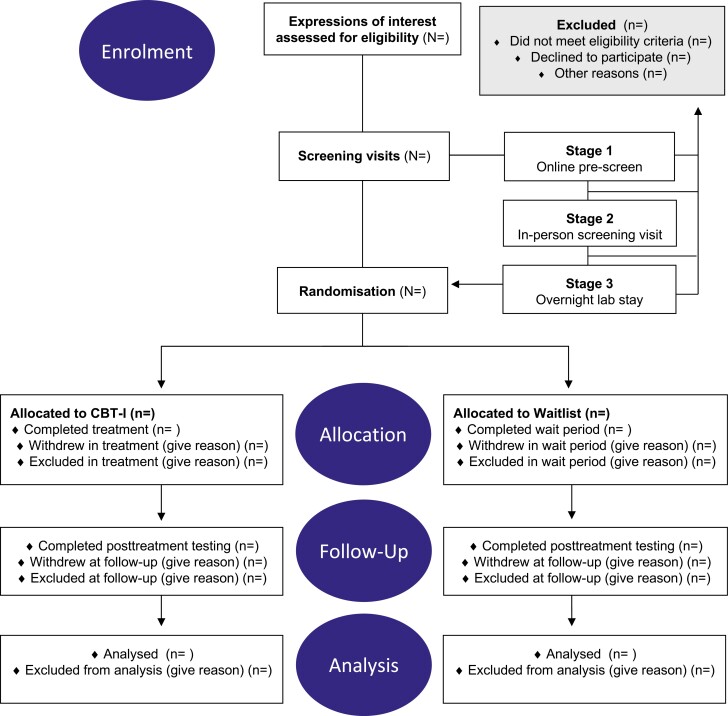
Flow Diagram for enrollment, allocation, follow-up, and analysis. CONSORT flow diagram outlining participant enrollment, allocation to condition, follow-up, disposition status, and analysis. *CBT-I cognitive behavioral therapy for insomnia*.

**Figure 2.**
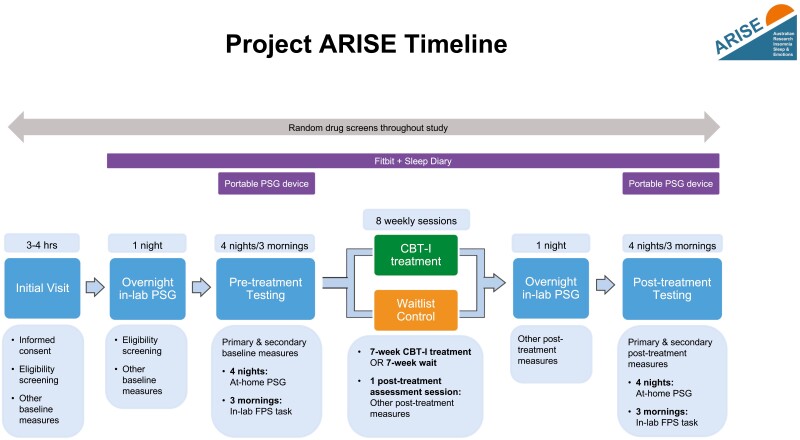
Study protocol timeline. Study protocol timeline outlining stages of screening, treatment, and pretreatment and post-treatment assessments. Participants are first screened via an initial 3–4 hours visit and overnight in-lab PSG. Eligible participants undergo a 4 night/3 morning pretreatment testing protocol, involving 4 nights of at-home PSG and 3 mornings of in-lab FPS testing. Participants then complete either 7 weeks of CBT-I or a 7-week wait period, followed by a post-treatment assessment session, a second in-lab PSG night, and a 4 night/3 morning post-treatment testing protocol identical to the first. Drug screens are administered throughout the study to ensure adherence to the requirement of drug abstinence. Participants wear a Fitbit and complete daily sleep diaries from the first in-lab PSG night until the end of post-treatment testing. *CBT-I cognitive behavioral therapy for insomnia, FPS fear-potentiated startle, PSG polysomnography.*

This randomized controlled study employs a 2 (group: active treatment vs. waitlist control) × 2 (time: pretreatment vs. post-treatment) mixed factorial design to examine the role of REM sleep in fear inhibition in a sample of individuals with insomnia disorder. Using block randomization, participants are randomly assigned in equal numbers to either an active treatment or waitlist control condition. Study assessment will occur at two main time points: once at baseline (pretreatment) and once within 2 weeks of completing either a 7-week treatment program for insomnia or a 7-week waitlist period (post-treatment). See Figure 1 for a flowchart of study enrollment, intervention, and assessments. A timeline of the study protocol is depicted in Figure 2. The study is preregistered on ANZCTR.

### Participants

A total of 92 adults with Insomnia Disorder will be recruited as part of a study on sleep, stress, and emotions (Project ARISE: Australian Research on Insomnia, Sleep, and Emotions). Participants are recruited via community advertisement (e.g. social media, radio, newspapers, Monash University websites), participant registries from sleep research institutes (e.g. Flinders Health and Medical Research Institute; Adelaide, Australia), and referrals from health care centers in Melbourne, Australia (e.g. Monash University Healthy Sleep Clinic). Taking into account data loss and attrition rates in past studies with similar designs, we expect to obtain 66 completers with useable data. Data loss are estimated based on prior repeated-measure fear inhibition studies where we observed approximately 25% data loss [19, 20] and an insomnia clinical trial where we observed 11% treatment drop-out [28].

### Inclusion and exclusion criteria

Participant eligibility criteria are outlined in Table 1 below.

**Table 1.** Eligibility Criteria

Inclusions
(a) Adults (>18 years of age). (b) Diagnosis of insomnia disorder according to DSM-5. (c) Fluent in English.
Exclusions
Sleep/circadian rhythm-related factors (a) Extreme chronotypes and unmanaged sleep disorders other than insomnia (e.g. obstructive sleep apnea with a global AHI ≥15; periodic limb movements disorder with a PLMA >10). Managed sleep disorders such as obstructive sleep apnea treated with continuous positive airway pressure is allowed. (b) Night or early morning shift work in the past 3 months. (c) Transmeridian travel (≥ 2 time zones) in the past 2 months (an adjustment period of 1 week per hour of time zone traveled is required before commencing the study). (d) Children <1 year of age as they impact sleep. (e) Behavioral treatment for insomnia within the past month. Psychiatric conditions (f) Personal history of PTSD or current Acute Stress Disorder. (g) Personal or family history of bipolar disorder, psychosis, or schizophrenia in first degree relatives. (h) >1 lifetime major depressive episode or current active episode of depression. (i) Current substance use disorder, eating disorder, panic disorder, or obsessive-compulsive disorder. (j) Personality disorders. Medical conditions (k) Cancer, uncontrolled diabetes or cardiovascular disease. (l) Chronic pain conditions (e.g. osteoarthritis, fibromyalgia, severe irritable bowel syndrome, migraines without aura ≥5 times/month, or migraines with aura ≥2 times/month). (m) All neurological conditions, history of stroke or acquired brain injury (mild concussions involving <15 minutes of loss of consciousness allowed). Drug use (n) Frequent cannabis use (>2 times/month) or other recreational drug use (>1 time/month) (o) Current use or recent discontinuation of medications known to affect REM sleep or fear inhibition (e.g. SSRIs/SNRIs, tricyclic antidepressants, opiates, orexin antagonists, benzodiazepines, hypnotics, corticosteroids, methylphenidate, amphetamine). Participants may be eligible if the drug has been safely discontinued for a minimum duration equivalent to 5 half-lives. Fear-potentiated startle task-related factors (p) Failure to exhibit a consistent startle response on day of screening (i.e. over 50% discernible responses to six 108-dB 40ms startle pulses). A normal, consistent startle response is required to measure fear inhibition via the fear-potentiated startle task we will use. (q) Age above 70 years, as this group has been shown to exhibit diminished startle responses and fear conditioning rates. (r) Failure to respond to a 35-dB pulse at 500, 1000 and 3000 Hz in both ears. Sex hormone-related factors (s) Current or recent discontinuation of treatments involving female sex hormones due to their impact on fear extinction processes (e.g., gender-affirming hormone treatments, fertility treatments). Hormonal birth control methods and hormone replacement therapies are allowed given dose remains stable throughout testing. (t) Currently pregnant or breastfeeding, or actively trying to conceive. (u) Hot flashes which are frequent (≥ 5/day) or cause significant interference with sleep (score ≥7 on sleep item in the Hot Flash Related Daily Interference Scale) In-treatment exclusions (v) Hospitalization. (w) Taking >10 weeks to complete the 7-week treatment program

DSM-5 diagnostic and statistical manual of mental disorders fifth edition, AHI apnea hypopnea index, PLMA, periodic limb movement arousal index; PTSD, posttraumatic stress disorder; SSRI, selective serotonin reuptake inhibitor; SNRI, serotonin–norepinephrine reuptake inhibitor.

### Screening and eligibility

Eligibility determination occurs in three stages. Firstly, upon expressing interest in the study, participants complete a brief prescreening questionnaire online or via telephone to assess initial eligibility. Questions cover major inclusion and exclusion criteria, including those related to their sleep, mental and physical health, and drug use.

Secondly, following written informed consent (obtained by trained research staff), participants engage in a 3- to 4-hour screening and pretreatment assessment in person. This assessment involves: (1) the Structured Clinical Interview for Sleep Disorders Revised (SCISD-R) [29] to assess insomnia and other potential sleep disorders; (2) the Structured Clinical Interview for DSM-5 (SCID-5) [30] to ascertain the presence of current and/or past psychiatric disorders, (3) a health interview to assess medical and sleep history, (4) a range of baseline self-report measures as listed in Table 2, (5) a urine drug test to confirm no disallowed drugs which may affect sleep are detectable prior to testing (participants are also screened randomly throughout the protocol), (6) a brief hearing test using an audiometer to assess whether participants can hear and respond to a 35-dB pulse at 500, 1000 and 3000 Hz in both ears, and (7) a startle test to confirm the participant’s startle response as measured by EMG of the blink reflex to six 108-dB 40ms startle pulses while seated in a sound-attenuated room. Thirdly, participants undergo overnight polysomnography (PSG) to screen for the presence of other unmanaged sleep disorders.

**Table 2.** Schedule of Enrollment, Intervention, and Assessments

	Study period												
	Enrollment		Allocation	Post-allocation							Close-out		
	Screening												
	Baseline			Intervention							Post-tx		
Timepoint	*-t2*	*-t1*	*0*	*t1*	*t2*	*t3*	*t4*	*t5*	*t6*	*t7*	*t8*	*t9*	*t10*
	*Surveys & Interviews*	In-lab PSG	Behavioral Testing								Surveys & interviews	In-lab PSG	Behavioral Testing
Enrollment													
Eligibility screen	X												
Informed consent	X												
Allocation			X										
Intervention													
CBT-I				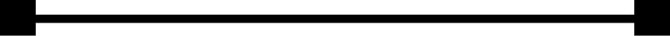									
Waitlist				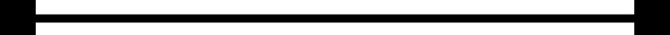									
Assessment													
Primary and secondary measures													
*REM fragmentation*													
At-home PSG (4 nights)			X										X
*Fear inhibition – extinction recall (primary); safety signal recall (secondary)*													
FPS task (3 mornings)			X										X
Serum estradiol (E2)			X										X
Other measures													
* Eligibility measures*													
Alcohol Use Disorders Identification Test (AUDIT)	X										X		
Drug Use Disorders Identification Test (DUDIT)	X										X		
Structured Clinical Interview for DSM-5 (SCID-5)	X												
Structured Clinical Interview for Sleep Disorders revised (SCISD-R)	X										X		
Hot Flash Related Daily Interference Scale (HFRDIS)	X												
*Daily measures*													
Sleep Diaries		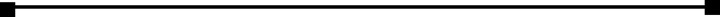											
Fitbit		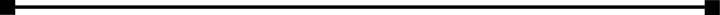											
*Treatment-related measures*													
Credibility Expectancy Questionnaire (CEQ)				X									
Client Adherence Form (CAF)					X	X	X	X	X	X			
Adverse Effects Scale					X	X		X		X	X		
Client Satisfaction Questionnaire (CSQ)											X		
*Other descriptive and exploratory measures*													
**Sleep-related Measures**													
Insomnia Severity Index (ISI)	X			X	X	X	X	X	X	X	X		
Epworth Sleepiness Scale (ESS)	X										X		
PROMIS Sleep-Related Impairment	X										X		
Sleep Hygiene Index	X										X		
Flinders Fatigue Scale (FFS)	X										X		
Ford Insomnia Response to Stress (FIRST)	X										X		
Dysfunctional Beliefs About Sleep (DBAS)	X								X				
Munich Chronotype Questionnaire (MCTQ core)	X										X		
Reduced Morningness Eveningness Questionnaire (MEQr)	X										X		
Presleep Arousal Scale	X	X					X				X	X	
Iowa Resistance to Sleeplessness Test	X												
Karolinska Sleepiness Scale (KSS)		X	X									X	X
*Mental health-related measures*													
Patient Health Questionnaire-9 (PHQ-9)	X										X		
PROMIS Anxiety – SF 8a	X										X		
Life Events Checklist for DSM-5 (LEC-5)	X										X		
PTSD Checklist-5 (PCL-5)	X										X		
PROMIS Satisfaction – SF 8a	X										X		
Warwick Edinburgh Mental Well-being Scale (WEMWBS)	X										X		
Brief Self-Control Scale (BSCS)	X												
Intolerance of Uncertainty Scale (IUS-12)	X												
Spielberger State-Trait Anxiety Inventory (STAI)		X	X									X	X
*Cognitive functioning-related measures*													
British Columbia Cognitive Complaints Inventory (BC-CCI)	X						X				X		
Paired Associates Learning (PAL)		X										X	

SPIRIT flow diagram outlining the schedule of enrollment, intervention, and assessments. CBT-I, cognitive behavioral therapy for insomnia; PSG, polysomnography.

### Randomization

Following screening assessments, participants are randomly assigned to either an active treatment or waitlist control condition, with a 1:1 allocation as per a computer-generated randomization schedule, stratified by sex (male vs. female), using permuted blocks. To ensure concealment, block size will not be disclosed. Allocation sequence is stored in a spreadsheet created by a researcher not actively involved in enrollment and assignment of participants to conditions. The sequence is concealed by making the cells opaque until a participant is screened, deemed eligible, and ready to be assigned to a condition. Then, the next cell in the sequence is revealed. To help reduce predictability of condition orders for study staff, men and women have separate randomization orders.

### Study intervention

#### Active treatment cognitive behavioral therapy for insomnia.

The active treatment group receives seven weekly sessions of cognitive behavioral therapy for insomnia (CBT-I) [31] These sessions are one hour each and delivered one-to-one with a trained therapist via telehealth (i.e. over Zoom). CBT-I is a multicomponent intervention targeting maladaptive behaviors and cognitions perpetuating the disorder. We use a manualized CBT-I protocol covering sleep restriction, stimulus control, sleep hygiene, relaxation techniques, cognitive restructuring, and relapse prevention. When commencing CBT-I, therapists and clients are provided a treatment manual to guide sessions and aid in the delivery of treatment. Client adherence to the intervention is monitored via daily sleep diaries, consumer sleep trackers, and a therapist-administered client adherence form (see Other Measures section). Concomitant behavioral and pharmacological treatments for insomnia will be prohibited for both active treatment and control groups. Client and therapist version of CBT-I manuals, sleep diary, and client adherence form are available here: https://osf.io/ecj64/?view_only=fa0f2e7f33bb445fb327b01b2ac5179a.

##### Therapists.

 Therapists are provisional or registered psychologists delivering treatment as part of their postgraduate placement in clinical psychology or paid employment. All therapists are supervised and trained in delivering CBT-I by study investigators (SPAD and BB) who are experienced clinicians and trainers in behavioral sleep medicine for the treatment of insomnia. Telehealth sessions are conducted by therapists either from the Turner Clinics at Monash University or at home. Risk assessments associated with telehealth, including home-based telehealth, are filed with Monash University. At least one supervisor is available remotely or on-site during each therapy session.

##### Treatment Fidelity.

 To ensure treatment fidelity, several factors will be implemented: (1) manuals are used in all treatment sessions and clinicians are given specific training on delivering the intervention (CBT-I) using the manual, (2) all treatment sessions are recorded over Zoom for fidelity checks in the delivery of treatment; (3) therapists receive weekly group supervision, which involves the supervisor watching selections of recordings from that week’s treatment, and (4) recordings from every session from the first two cases of each trained therapist and 14% of all subsequent sessions (1 session/participant) are evaluated for treatment fidelity based on a standardized checklist of session content (see https://osf.io/ecj64/?view_only=fa0f2e7f33bb445fb327b01b2ac5179a). Fidelity checklists will be completed by a trained clinical psychologist who is experienced in delivering manualized CBT-I. The 14% of all subsequent sessions undergoing fidelity checks will be counterbalanced, to ensure equal representation across all 7 sessions of treatment for each therapist.

#### Waitlist control.

The control group is informed they have been placed on a waitlist for treatment and will undergo a waiting period for the duration of 7 weeks. Waitlist participants are then offered the same 7-week course of CBT-I upon completion of the study protocol. Although a waitlist condition has less face validity than a placebo or active control condition, it will be used to minimize the risk of affecting REM sleep in the comparator group.

### Assessment timing

As depicted in Figure 2, there are three types of study assessments: (1) primary and secondary measures, (2) daily assessments of sleep, and (3) other descriptive and exploratory measures. Primary and secondary outcome measures (i.e. fear inhibition) are assessed in a 4 night/3 day behavioral testing protocol before and after completing either the 7-week CBT-I treatment or a 7-week waiting period. To control for the known effects of female sex hormones on fear extinction [32–34], Fear-Potentiated Startle testing (see Fear Inhibition assessment, below) will be scheduled during the high estradiol (i.e. late follicular) phase of the menstrual cycle where possible. Daily sleep measures are collected via sleep diary and consumer sleep tracker throughout the study. The other descriptive and exploratory measures are assessed at pre- and post-treatment (see other measures: other descriptive and exploratory measures, below).

### Primary and secondary measures

**Figure 3.**
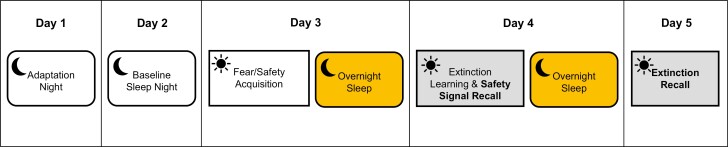
Pre- and post-treatment testing protocol. Pre- and post-treatment testing protocol involving 4 nights of at-home PSG and 3 mornings of in-lab FPS testing. The first night serves as an adaptation night and the second night serves as a measure of baseline sleep. The following three mornings, participants complete the fear and safety acquisition phase, extinction learning and safety recall phase, and extinction recall phase of the FPS protocol, respectively (See Figure 4). *FPS fear-potentiated startle, PSG polysomnography.*

REM sleep and fear inhibition will be assessed using a 4 night/3 day testing protocol (see Figure 3) as outlined below.

#### REM sleep.

REM sleep is assessed at home for four consecutive nights using a portable PSG device, the Dreem 3 Ambulatory Electroencephalography (EEG) system (Dreem™, France, Paris). Night 1 serves as an adaptation night, Night 2 serves as a baseline night, and nights 3 and 4 serve as the experimental nights. The Dreem 3 device is a wireless headband worn during sleep. Two main types of sensors in the device are used to record sleep-related physiological data: (1) five dry EEG electrodes (F7, F8, O1, O2, and Fp2) yielding five derivations (F7-O1, F8-O2, F8-F7, F8-O1, and F7-O2) recording cortical activity in the brain, (2) a 3D accelerometer records movement, respiratory rate/trace, and head position. Signal acquisition and the automatic algorithm for sleep staging used by the device have been shown to be comparable to standard PSG scored by expert sleep technicians [35]. We will utilize the automatic scoring algorithm to identify sleep stages. In those cases where signal quality is not sufficient for automated scoring (signal quality is an output of the algorithm), two trained technicians will manually score the PSG. REM sleep data recorded from experimental nights 3 and 4 will be used to test our hypotheses.

#### Fear inhibition

**Figure 4.**
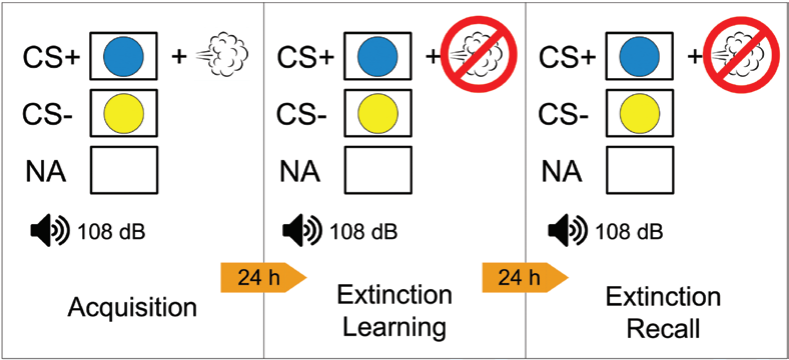
FPS protocol. FPS protocol used in the current study involves 3 consecutive days of testing. Day 1 (Fear/Safety Acquisition) includes 8 presentations of the CS+ (e.g. blue circle) paired with an air puff to the larynx 75% of the time, 8 presentations of the CS- (e.g. yellow circle) never paired with the air puff, and 12 NA trials where the 108-dB startle probe is delivered in the absence of any visual cue. Day 2 (Extinction Learning) includes 16 CS+ and 16 CS- presentations, and 20 NA trials. However, no air puffs are delivered in this session. Day 3 (extinction recall) includes 8 CS+ and 8 CS- presentations, and 12 NA trials. Again, no air puffs are delivered in this session. *CS+ conditioned fear cue, CS- conditioned safety cue.*

After baseline night (night 2), fear inhibition is assessed using the fear-potentiated startle (FPS) task over three consecutive mornings on days 3–5 (see Figure 3). The FPS task is a well-validated paradigm that relies on the acoustic startle reflex to measure conditioned fear. The task is sensitive to PTSD symptoms as well as manipulations of sleep [6, 24] Testing sessions occur 2 hours following participants’ habitual wake time, as fear extinction is strongest in the morning [36]. To take into account effects of state anxiety and sleepiness on task responses, participants complete the Karolinksa Sleepiness Scale (KSS) [37] immediately before each testing session, and the State-Trait Anxiety Inventory (STAI) [38] immediately before and after each session. For all sessions, participants are seated in a sound-attenuated room and visual cues are presented via an LCD monitor. Two Ag/AgCl electrodes are positioned on the left orbicularis oculi muscle (approximately 1cm below the pupil and 1cm below the lateral canthus), and a ground electrode is placed on the left mastoid. These sensors are fitted to measure electromyography (EMG) of the blink reflex to startling tones. EMG data sampling rate is 1 KHz. Data are amplified from 0.5 mV electrode input to 2500 mV signal output, band-pass filtered (100–1000 Hz), rectified, and smoothed with a 5-point rolling average. These EMG data serve as operational measures of conditioned fear, safety learning, safety recall, extinction learning, and extinction recall. Fear inhibition is operationalized as safety recall and extinction recall. The FPS task involves three phases (see Figure 4).

#### Morning 1: fear/safety acquisition.

In the first session, participants acquire conditioned fear to a visual cue (conditioned stimuli, CS+). A mildly aversive 275-280 PSI puff of air to the larynx serves as the unconditioned stimulus (US). Testing begins with a 2-minute acclimation period followed by 3 types of trials in pseudo-randomized order across 8 blocks: 2 conditioned stimulus (CS) trials and 1 no-stimulus trial. In each CS trial, participants are presented with 1 of 2 possible shapes for 6 seconds. One shape (e.g. a blue circle) is paired with the air puff 75% of the time and serves as the fear-conditioned stimulus (CS+). The other shape (e.g. yellow circle) is never paired with the aversive puff and serves as the safety signal (CS−). Participants are not explicitly informed of the predictive quality of these cues but are asked to report air puff expectancy (“yes,” “no,” or “unsure”) for each trial to track their CS+/CS− contingency learning. Between 4 and 5 seconds after the onset of each shape, a startle probe (108-dB 40 milliseconds burst of white noise) is delivered binaurally via headphones to assess fear conditioning. In the no-stimulus trial, the startle probe is presented in the absence of any visual cue (i.e. noise alone [NA)] to get a measure of baseline startle. Fear-potentiated startle is operationalized as eyeblink magnitude in response to the startle probe when presented in the presence vs. absence of the CS+ (i.e. CS+ trials—NA trials). Fear Conditioning will be assessed as the degree of startle potentiation (vs. NA trials) in the presence of the CS+, relative to startle potentiation in the presence of the CS− in the last block of trials. Safety Signal Learning will be assessed by calculating changes in startle amplitude to the CS− from the first block (when participants typically startle to all stimuli), to the last block (when they have learned the CS− predicts safety) [19]. To minimize practice effects, different visual cues will be used at each time point (pre- and post-treatment) and order will be counterbalanced.

#### Morning 2: extinction acquisition/safety signal recall.

The second session involves extinction learning and a test of safety signal recall. Participants are presented with the same visual cues and startle probes as the first session, but no air puffs are delivered. Thus, participants learn the fear cue (CS+) no longer predicts the aversive stimulus (US), and the conditioned fear response is extinguished. All other procedures are the same as above. Extinction learning is assessed as the change in startle response to the fear cue (CS+) over the extinction session. Safety signal recall (the variable of interest for our aims) is operationalized as the change in startle response to the safety cue (CS−) from the last block of session 1, to the first block of session 2.

To statistically control for residual differences in estradiol among non-naturally cycling participants (e.g. hormonal contraception users, post-menopausal participants), participants of female sex will also provide a blood serum sample on the morning of extinction acquisition, to assess for serum estradiol levels given their role in extinction retention [39].

#### Morning 3: extinction recall.

The third session is identical to session 2 and serves as a test of extinction retention. Consistent with prior research using this paradigm [24, 40, 41], extinction recall (the variable of interest for our aims) is operationalized as the change between the maximal startle response to the CS+ during acquisition in session 1 and the average startle response to the CS+ over the first block of extinction recall. Greater differences between CS+ responses during acquisition and extinction recall suggest stronger extinction recall [40].

### Other measures

All other measures will be administered to screen for participant eligibility, characterize the sample, and/or support exploratory analyses.

#### Eligibility measures.

The following are administered at the initial in-person screening visit.

##### Alcohol Use Disorders Identification Test.

[42] A self-report questionnaire designed to identify individuals with hazardous and harmful patterns of alcohol consumption.

##### Drug Use Disorders Identification Test.

[43] A self-report questionnaire designed to identify individuals with harmful drug-related problems.

##### Structured Clinical Interview for DSM-5.

[44]: A structured interview designed to assess for psychiatric disorders according to criteria from the DSM-5. Only specific modules relevant to this study’s eligibility criteria are administered (see exclusions [f–j]in Table 1).

##### Structured Clinical Interview for Sleep Disorders, revised version.

[45] A semi-structured interview designed to assess for the most common sleep disorders using criteria from the DSM-V. Responses are used to confirm that participants meet criteria for a diagnosis of Insomnia Disorder and do not meet criteria for any other unmanaged sleep disorders. Unlike the other eligibility measures, the SCISD is also readministered at post-treatment to assess whether participants still meet criteria for Insomnia Disorder.

##### Hot Flash Related Daily Interference Scale.

[46] A self-report measure assessing the impact of hot flashes on overall quality of life and nine specific activities. Only one item related to impact on sleep will be administered for eligibility purposes.

#### Daily measures.

Once participants are deemed eligible, the following measures are administered daily for the duration of the study to assess sleep/wake patterns before, during, and after treatment (see Figure 2).

##### Sleep Diaries.

Each morning, participants record sleep habits in a sleep diary based on Carney et al.’s (2012) consensus sleep diary [47]. Measures include bed and wake time, time in bed, number and duration of awakening and naps, sleep latency, final time they got out of bed, and three questions to assess adherence to Stimulus Control Therapy. The diary automatically calculates total sleep time, minutes spent awake after sleep onset, sleep opportunity (i.e. time-in-bed), and sleep efficiency.

##### Fitbit.

A wrist-worn activity tracker (Fitbit Charge 5™; Fitbit Inc.) will be used to monitor sleep–wake patterns throughout the study.

#### Treatment-related measures.

The following measures are administered at various timepoints throughout treatment (see Table 2 for schedule of administration).

##### Credibility Expectancy Questionnaire.

[48] A self-report measure assessing treatment expectancy and perceived rationale credibility.

##### Client Adherence Form.

[28] A therapist-administered measure based on the Patient Adherence Form used by Trockel et al. (2014) [49] to gauge the individual’s adherence to 7 specific CBT-I activities (e.g. completing sleep diaries, stimulus control, etc.) each week.

##### Adverse Effects Scale.

 [50] A self-report scale designed to assess adverse events and symptoms experienced as a result of CBT-I treatment.

##### Client Satisfaction Questionnaire.

 [51] An 8-item questionnaire that provides a measure of client satisfaction with services received.

#### Other descriptive and exploratory measures.

The following sleep, mental health, and cognition-related measures are administered at baseline (i.e. pretreatment) and post-treatment (see Table 1 for schedule of administration). At baseline, they are typically administered at the initial screening visit and/or the first overnight in-lab PSG. However, we require all measures other than the eligibility measures to be completed within one month of the baseline FPS testing session. Therefore, if there is a predictable delay in that test session (e.g. due to scheduling around menstrual cycle or work demands), we administer the baseline questionnaires during the week prior to the FPS test session. At post-treatment, they are typically administered at the post-treatment assessment session and/or the second overnight in-lab PSG.

### Sleep-related measures

#### Insomnia Severity Index.

 [52] A 7-item self-report measure assessing the nature, severity, and impact of insomnia in the past week.

#### Epworth Sleepiness Scale.

 [53] An 8-item self-report measure assessing the likelihood of an individual to fall asleep in certain situations, yielding an overall measure of daytime sleepiness.

#### PROMIS sleep-related impairment – short form 8a.

[54] An 8-item self-report assessment tool measuring the impact of poor sleep on daytime activities.

#### Sleep hygiene index.

 [55] A 13-item self-report scale measuring frequency of sleep hygiene behaviors, with items derived from the International Classification of Sleep Disorders diagnostic criteria for inadequate sleep hygiene.

#### Flinders fatigue scale.

 [56] A 7-item self-report scale measuring various characteristics of daytime fatigue experienced over the past 2 weeks.

#### Ford insomnia response to stress test.

 [57] A self-report measure of sleep reactivity to assess an individual’s vulnerability to insomnia under stress.

#### Dysfunctional beliefs about sleep.

 [58] A 16-item self-report measure assessing individuals’ beliefs and attitudes about sleep.

#### Munich chronotype questionnaire – core.

 [59] A shortened version of the standard MCTQ containing only the core chronotype module of the original questionnaire, designed to determine an individual’s chronotype by assessing their sleep behavior.

#### Reduced morningness–eveningness questionnaire.

 [60] A reduced, 5-item version of Horne & Östberg’s Morningness–Eveningness questionnaire, designed to determine an individual’s chronotype by assessing their diurnal preference.

#### Presleep arousal scale.

 [61] A 16-item self-report scale measuring cognitive and somatic manifestations of arousal at bedtime.

#### Iowa resistance to sleeplessness test.

 [62] A 16-item self-report measure assessing individuals’ resistance to the cognitive and affective consequences of moderate sleep **loss.**

#### The karolinska sleepiness scale.

[37] The KSS consists of 1 self-report item pertaining to perceived level of sleepiness.

### Mental Health-related Measures.

#### Patient health questionnaire-9.

 [63] A 9-item questionnaire assessing severity and frequency of depression symptoms.

#### PROMIS anxiety - short form 8a.

[64] An 8-item questionnaire assessing severity of anxiety symptoms.

#### Life events checklist for DSM-5.

[65] A self-report measure designed to screen for potentially traumatic events in a respondent’s lifetime. This is administered prior to the SCID-5 to inform the clinician if they should complete the PTSD module.

#### Posttraumatic stress disorder checklist-5.

 [66] A questionnaire assessing trauma symptoms based on the DSM-5. This is administered prior to the SCID-5 to inform the clinician if they should complete the PTSD module.

#### PROMIS satisfaction with social roles and activities - short form 8a.

[67] An 8-item questionnaire assessing satisfaction with performing one’s usual social roles and activities.

#### Warwick edinburgh mental well-being scale.

 [68] A 14-item questionnaire that provides a measure of mental well-being, focusing entirely on positive aspects of mental health.

#### Brief self-control scale.

 [69] A 13-item self-report measure assessing individual differences in self-control.

#### Intolerance of uncertainty short form.

 [70] A 12-item self-report questionnaire to measure responses to uncertainty, ambiguous situations, and the future.

#### Spielberger state-trait anxiety inventory.

[71] The STAI consists of 40 self-report items pertaining to anxiety. To assess presleep arousal, only questions relating to state anxiety will be administered at bedtime on in-lab PSG nights at pre- and post-treatment.

### Cognitive functioning-related measures

#### British Columbia cognitive complaints inventory.

 [72] A 6-item self-report measure assessing perceived cognitive problems (concentration, memory, thinking skills).

#### Paired associates learning task.

The paired associates learning task is a computerized test of episodic associative memory. In this study, we will use a version suited for measuring overnight memory consolidation ability. The task comprises 48 semantically related single-word pairs and consists of three phases, two in the evening and one the following morning. In the initial learning phase, each word pair is sequentially presented on a computer screen. After all the word pairs are presented, the first word of each word pair is presented in random order to the participant. The participant is instructed to respond with the corresponding word which makes the word pair. This process (i.e. presentation of word pairs and immediate testing) is repeated until the participant correctly recalls 60% or more-word pairs on immediate testing. The next phase occurs after a short delay (30 minutes after the learning phase) and comprises a testing phase whereby the first word from each word-pair is presented and the participant is instructed to respond with the corresponding word. The final morning testing phase will be identical to the evening short delay testing phase but will occur in the following morning approximately 30 minutes after the participant awakens. Data from this task will be used for a separate study related to the effects of CBT-I on cognition in comorbid insomnia and obstructive sleep apnea.

### Blinding

Participants are blind to study hypotheses and outcome variables. Post-treatment assessments involving clinical judgment (e.g. SCISD) are conducted by assessors who are blind to treatment condition, and participants are explicitly asked and reminded not to mention or reveal their treatment condition to these assessors. Additionally, polysomnography and FPS data are also scored by researchers who are blind to treatment conditions.

### Participant retention

To promote participant retention and minimize data loss, participants receive weekly check-ins regarding their progress with daily sleep diaries, are reminded of upcoming data collection points, and are offered greater reimbursement for completing the FPS testing protocol at post-treatment than pretreatment. Reasons for participant non-retention will be recorded (e.g. consent withdrawn, lost to follow-up).

### Data collection and management

Data will be deidentified wherever possible, and information from potential and enrolled participants will be collected, entered, and stored electronically in a secure database only accessible by researchers. Data from any hardcopy materials with participant data (e.g. therapist-administered SCID and SCISD interview forms) will be entered and stored securely at Monash University. Participant data were only be shared outside the research team with the participant’s written consent (e.g. when referring excluded participants to external clinics). Only investigators and key research staff involved in data processing and analysis will have access to the final dataset. Participant data will be maintained for a period of 15 years after completion of the study, unless written consent for use in future studies is obtained.

### Data processing

#### Fear inhibition measures.

FPS data were first be cleaned of movement artifacts and voluntary eye blinks, trial-by-trial, based on visual examination of a window from 100 milliseconds before pulse onset to 100 milliseconds after pulse onset, conducted by a blind rater. Only responses peaking within 100 milliseconds of pulse onset are scored. Trials with excessive baseline noise or artifacts before or during the trial are removed. Data for those trials are imputed based on the average value of the immediately preceding and following trials. Data were then analyzed by averaging peak responses to each stimulus type within a block. Baseline startle-to-NA trials will be used to determine potentiated startle responses, and values for extinction and safety recall at the two-time points will be calculated, as defined above. Should group differences in overall startle activity preclude the ability to interpret extinction effects, data were transformed to individual z-scores within each session to reduce overall variability in baseline response [20].

#### REM sleep latent variable.

At-home PSG data will be scored according to the American Academy of Sleep Medicine guidelines. Based on the findings from Marshall et al. (2014) and Straus et al. (2017), a REM Sleep latent variable will be calculated from three REM variables: REM% (REM sleep duration/total sleep duration), REM efficiency (number of REM minutes/total duration of all REM periods), and REM latency (duration of non-REM sleep before the first REM onset). Lower REM%, lower REM efficiency, and shorter REM latency will be used as indices of greater REM fragmentation.

#### Serum estradiol (E2).

Serum will be processed from 5 to 10 mL of blood sampled via venepuncture immediately after morning 2 of the FPS task (extinction training). Blood samples are left undisturbed at room temperature for 30–60 minutes to coagulate before centrifugation (3000RPM, 20–25 degrees Celsius) for 10 minutes or until serum has sufficiently separated from clotted packed cells. Serum is then pipetted into 0.5 mL aliquots, stored at −78–81 degrees Celsius, and assayed for estradiol via liquid chromatography–mass spectrometry (LCMS).

### Power considerations

No studies have examined the relationship between REM sleep and fear inhibition in an insomnia sample, nor have any attempted to reverse REM disruption to improve fear inhibition. Nonetheless, several studies from our team have demonstrated REM sleep-fear inhibition relationships in healthy controls and individuals with PTSD [19, 20, 24], with effect sizes ranging from *r* = 0.318 to *r* = 0.688 (medium to very large, per Cohen [73]), with a sample-size weighted average effect size of *r* = 0.477. Since that value is skewed towards healthy control studies, we will use an estimated effect size here of *r* = 0.40 (halfway between medium and large) for Aim 1 and a parallel effect size for Aim 2. Based on *r* = 0.40 and *α* = 0.05, *n* = 62 participants will provide power of 0.90 in Aim 1. For Aim 2, *f* = 0.33 (also medium-large) and *α* = 0.05, *n* = 66 provides power of >0.95 for Hypothesis 2a and >0.80 for the mediation analysis (Hypothesis 2b) [74].

### Data analysis plan

All analyses will be conducted on an intention-to-treat basis. To address missing data, multiple imputation [75] will be used for regression-based analyses, and full information maximum likelihood [76] will be used for mediation analyses. Independent samples t-tests will first be used to examine potential differences in demographic variables between the treatment and control groups (e.g. age, sex, and ISI), whilst controlling for family-wise error rates.

*Aim 1*

Hypothesis 1: REM sleep predicts fear inhibition performance the next day. Two simple linear regression analyses will be conducted to assess how well REM fragmentation (i.e. low values on the REM Sleep latent variable) the night before predicts extinction and safety recall the following day. Baseline REM fragmentation on night 3 will be used as a predictor for level of safety signal recall the next day. Similarly, baseline REM fragmentation on night 4 will be used as a predictor for level of extinction recall the next day. The analyses will include parameters for sex and serum estradiol as covariates. In separate exploratory analyses, average REM sleep over the prior 3 or 4 nights, respectively, and each of the three REM variables will also be examined as explanatory variables. Finally, we will run sensitivity analyses utilizing baseline ISI scores as the explanatory variable to further examine the overall hypothesis that REM sleep abnormalities in Insomnia Disorder, rather than insomnia severity more generally, affect fear extinction and safety signals.

*Aim 2*

Hypothesis 2(a): Treating insomnia enhances fear inhibition. A linear mixed model will be used to examine the effect of treatment exposure (i.e. condition) on fear inhibition (i.e. extinction recall and safety recall). The model will include parameters for treatment condition, baseline fear inhibition, sex, and serum estradiol as a covariate.

Hypothesis 2(b): Treatment effects on fear inhibition are driven by improvements in REM sleep specifically. To assess the degree to which treatment-related changes in fear inhibition are mediated by improvements in REM sleep, three separate bootstrap mediation analyses will be used. The first model will include the direct effect of treatment group (CBT-I vs. waitlist) on changes in fear inhibition, and changes in the REM Sleep latent variable (pre- to post-treatment) as a mediator variable. Identical models will then be employed to examine whether changes in slow wave sleep (N3%) or overall insomnia severity (ISI) are related to the changes in fear inhibition. This approach will allow us to assess whether treatment effects are mediated by changes specific to REM sleep, rather than general improvements in sleep. For the mediation analyses, bootstrapped confidence intervals will be used to test statistical significance of indirect effects. Mediation analyses will be carried out in Mplus [77], with statistical significance of indirect effect determined using 95% bootstrapped confidence intervals from 20 000 resamples. In an exploratory analysis, each of the three REM variables will also be examined separately as potential mediators.

### Monitoring harms

During CBT-I, participants are informed of potential difficulties they may experience as part of the sleep restriction component (e.g. daytime sleepiness, fatigue, irritability), and potential risks for adverse events are assessed and discussed with participants to ensure preventative management strategies are put in place (e.g. weekly check-ins, not driving or operating heavy machinery during these periods if experiencing daytime sleepiness). Participants undergoing CBT-I will complete the adverse events scale to report negative side effects, and this information will be discussed in session, as needed. Additionally, participants in either condition will discontinue treatment and/or be withdrawn from the study should they be hospitalized for any reason throughout their participation, or for the emergence of (hypo)manic or psychotic symptoms sufficient to require clinical attention.

### Publications

All results will be published and communicated to interested participants via email. Publications will include those related to both the main aims and hypotheses of the study (primary and secondary outcomes), as well as those derived from exploratory analyses.

## Discussion

This study will be the first to test an underlying mechanism (REM sleep) that could explain why insomnia significantly increases one’s risk for PTSD. If our hypotheses are borne out, our findings will have several significant clinical implications. Firstly, we will have identified a modifiable risk factor addressable before trauma exposure in high-risk populations (e.g. emergency service workers, ICU staff), as well as quickly treatable immediately after trauma exposure. Moreover, we will have shown a gold-standard clinical intervention directly impacting REM disruptions (CBT-I) can influence the critical fear-related processes necessary to reduce the incidence of PTSD by promoting natural recovery from acute trauma symptoms. This would be extremely significant, as currently, very few clinical interventions can reliably reduce the incidence of PTSD after trauma exposure. Secondly, if CBT-I successfully improves fear inhibition by improving REM sleep, the use of other target methods to improve REM sleep may be warranted in specific populations. For instance, dual orexin receptor antagonists might be useful for short-term use in the immediate aftermath of a trauma, given their ability to improve REM sleep in insomnia [78]. Thirdly, if the REM sleep mechanism we test is correct, then other conditions characterized by REM disruptions may also be associated with impaired fear inhibition and these individuals may also benefit from preventative interventions to reduce the risk of PTSD. Given that REM disruption is seen in several other conditions (e.g. OSA, circadian disorders, jet lag, shift work, substance abuse, several psychiatric disorders, etc.), we will have potentially identified a transdiagnostic mechanism conferring increased risk of PTSD in a very large subset of the population. Finally, given the role of fear inhibition in most other anxiety disorders, targeting REM sleep may have implications for reducing the risk of not only PTSD, but a broad array of anxiety disorders.

### Limitations

One major limitation of the current study involves the generalizability of the findings given the extensive exclusion criteria for participant eligibility. Although involving randomization to an intervention, this study is at its heart a mechanism study as our primary outcomes of interest are experimental (i.e. REM fragmentation and fear inhibition) rather than clinical variables. As a result, maximizing internal validity and minimizing potential confounds and noise in our data was considered more pertinent than maximizing external validity. A second limitation involves the lack of an active control condition. Active control conditions such as sleep hygiene therapy were considered but avoided due to the lack of evidence evaluating whether these conditions affect REM sleep. Furthermore, we are not examining the efficacy of CBT-I (which is already well-established), but rather using the intervention to manipulate REM sleep. Thus, a waitlist control condition was considered sufficient for our purposes and least likely to affect our measures of REM sleep. Thirdly, ambulatory PSG measurement of REM sleep carries some risk of missing data or poor signal quality that could otherwise have been corrected using in-lab PSG measures. However, given insomnia participants often sleep better in the lab than at home [26] (possibly due to factors related to conditioned arousal), studying participants’ sleep in a more natural environment offers a more valid and accurate assessment of our main outcome variable. To minimize data loss and poor signal quality, a number of measures will be taken: (1) researchers will provide participants with one-to-one training on the correct positioning and use of the device, and ways to enhance signal quality (including providing them with a sweatband validated by Dreem to enhance the fit of the device), (2) participants will be provided with feedback and support on signal quality after the first adaptation night, to minimize data loss on subsequent testing nights, and (3) participants will have access to after-hours support from researchers on these nights for troubleshooting any problems encountered.

## Data Availability

The treatment manual, sleep diaries, and other measures developed by the study team are freely available at: https://osf.io/ecj64/?view_only=fa0f2e7f33bb445fb327b01b2ac5179a

## Author Credit Taxonomy

Vivien Vuong (Investigation [Lead], Methodology [Supporting], Project administration [Supporting], Visualization [Lead], Writing – original draft [Lead], Writing – review & editing [Lead]), Alix Mellor (Investigation [Supporting], Methodology [Supporting], Project administration [Lead], Writing – review & editing [Supporting]), Victoria Risbrough (Conceptualization [Supporting], Formal analysis [Equal], Funding acquisition [Supporting], Methodology [Supporting], Resources [Supporting], Visualization [Supporting], Writing – review & editing [Supporting]), Bei Bei (Conceptualization [Supporting], Formal analysis [Equal], Funding acquisition [Supporting], Methodology [Supporting], Resources [Supporting], Writing – review & editing [Supporting]), and Sean Drummond (Conceptualization [Lead], Formal analysis [Equal], Funding acquisition [Lead], Methodology [Lead], Resources [Lead], Supervision [Lead], Visualization [Supporting], Writing – review & editing [Supporting]).
